# Association between malaria immunity and pregnancy outcomes among Malawian pregnant women receiving nutrient supplementation

**DOI:** 10.1186/s12936-016-1597-7

**Published:** 2016-11-09

**Authors:** Upeksha P. Chandrasiri, Freya J. I. Fowkes, James G. Beeson, Jack S. Richards, Steve Kamiza, Kenneth Maleta, Per Ashorn, Stephen J. Rogerson

**Affiliations:** 1 Department of Medicine at the Doherty Institute of Infection and Immunity, University of Melbourne, 792 Elizabeth Street, Melbourne, VIC Australia; 2 Macfarlane Burnet Institute of Medical Research, 85 Commercial Road, Prahran, VIC Australia; 3 Department of Epidemiology, Centre for Epidemiology and Biostatistics, University of Melbourne, Melbourne, VIC Australia; 4 Department of Preventive Medicine and Infectious Diseases, Monash University, Clayton, VIC Australia; 5 Department of Microbiology, Monash University, Clayton, VIC Australia; 6 University of Malawi College of Medicine, Blantyre, Malawi; 7 Faculty of Public Health and Family Medicine, University of Malawi College of Medicine, Blantyre, Malawi; 8 Centre for Child Health Research, University of Tampere School of Medicine and Tampere University Hospital, Tampere, Finland; 9 Department of Paediatrics, Tampere University Hospital, Tampere, Finland

**Keywords:** Malaria in pregnancy, Malawi, Pregnancy outcomes, Variant surface antigens, Merozoite antigens, Anaemia, Birthweight, Small for gestational age, Placental malaria and low length-for-age Z score

## Abstract

**Background:**

Malaria antibody responses measured at delivery have been associated with protection from maternal anaemia and low birth weight deliveries. Whether malarial antibodies present in the first half of pregnancy may protect from these or other poor birth outcomes is unclear. To determine whether malaria antibodies in the first half of pregnancy predict pregnancy outcomes, antibodies were measured to a range of merozoite antigens and to antigens expressed on the surface of parasitized red blood cells (pRBCs) in plasma samples collected at 14–20 weeks of gestation from Malawian women. The latter antibodies were measured as total IgG to pRBCs, and antibodies promoting opsonic phagocytosis of pRBCs. Associations between antibodies and maternal haemoglobin in late pregnancy or newborn size were investigated, after adjusting for potential covariates.

**Results:**

Antibodies to pRBC surface antigens were associated with higher haemoglobin concentration at 36 weeks. Total IgG to pRBCs was associated with 0.4 g/l [(95% confidence interval (0.04, 0.8)] increase in haemoglobin, and opsonizing antibody with 0.5 (0.05, 0.9) increase in haemoglobin for each 10% increase in antibody. These antibodies were not associated with birthweight, placental malaria, or newborn anthropometrics. Antibodies to merozoite antigens and non-placental-binding IEs were not associated with decreased risk of any of these outcomes. In some instances, they were negatively associated with outcomes of interest.

**Conclusion:**

Antibodies to placental-binding infected erythrocytes may be associated with higher haemoglobin levels in pregnancy, whereas antibodies to other malaria antigens may instead be markers of malaria exposure.

*Trial registration* clinicaltrials.gov NCT01239693. Registered Nov 10, 2010.

## Background

Malaria in pregnancy accounts for an estimated 10,000 maternal and 200,000 infant deaths per year worldwide with over 25 million pregnancies at risk of infection in sub-Saharan Africa alone [1], and is predominantly caused by *Plasmodium falciparum*. Many of the associated deaths are due to severe maternal anaemia and low birth weight (LBW) [2]. Peripheral blood *P. falciparum* infections generally peak between 13 and 18 gestation weeks, which is when parasitized red blood cells (pRBCs) begin to sequester in the placenta [3, 4]. Placental adherence of pRBCs is mediated by the interaction between the pregnancy-related variant surface antigen (VSA) VAR2CSA expressed on the surface of the pRBC and chondroitin sulphate A (CSA) receptors expressed by the placental syncytiotrophoblast [5, 6]. With repeated pregnancies, women acquire antibodies to pRBCs that express VAR2CSA and develop protection against infection [7, 8], that is associated with improved pregnancy outcomes [9, 10]. By contrast, antibodies to non-pregnancy-related malaria antigens (merozoite antigens, schizonts and non-pregnancy-specific VSA) have shown little association with pregnancy outcomes [11–13].

Women who are parasitaemic at first antenatal care visit may have an increased risk of adverse outcomes such as LBW, placental infection, preterm birth and anaemia [14–17]. It is possible that measures of malaria immunity at this point could be used to identify women at relatively high risk of poor pregnancy outcomes. Most studies to date but not all [18–20] have investigated the relationship between immunity measured at or close to delivery with pregnancy outcomes. While relevant to defining the potential protective role of malaria immunity in pregnancy outcomes, such studies would not allow health workers to identify women at high or low risk of such outcomes in a timely way, to allow them to offer tailored malaria prevention strategies. If only a proportion of pregnant women are at high risk, and these women could be identified at first antenatal attendance, malaria preventive strategies could be targeted to those lacking protection and at greatest need.

Acquisition of antibodies is dependent on a number of factors such as maternal age, gravidity and presence of other infections such as HIV [7, 21, 22]. In addition, malaria infection, HIV and gravidity have been shown to modify the effects of the association between malaria immunity and pregnancy outcomes [12, 23]. Malaria prevention strategies such as indoor residual spraying, insecticide-treated bed nets (ITNs) and intermittent preventive treatment in pregnancy (IPTp) are designed to reduce the exposure to malaria and improve pregnancy outcomes, but may impair acquisition of immunity [24]. These factors must be considered when determining the relationship between malaria immunity and pregnancy outcomes.

Pregnant women residing in malaria-endemic regions often suffer from undernutrition as a result of poverty, seasonal variation in food availability and poor intake of micronutrients, which significantly impact their health and pregnancy outcomes. It was hypothesized that among Malawian women enrolled in a nutrient supplementation study, one or more measures of naturally acquired malaria antibody, taken at first antenatal visit, would be associated with protection from adverse pregnancy outcomes such as maternal anaemia, evidence of malaria on placental histology, or measures of fetal growth in utero.

## Methods

### Study context

From February 2011 to August 2012 one thousand three hundred and ninety-one pregnant women were enrolled in the iLiNS-DYAD-M randomized controlled trial, which evaluated whether home fortification of pregnant women’s diets with nutrient supplements could increase birth size. Participants received either iron and folic acid supplements, a multiple micronutrient capsule, or 20 g of lipid-based nutrient supplementation each day from enrolment, from 14 to 20 completed weeks of gestation, until delivery. Dating ultrasounds were performed at enrolment to determine gestation at delivery. All participants received 2 doses of sulfadoxine-pyrimethamine (SP, Fansidar^®^). The trial design, details of the nutrient supplements, and main findings have been published elsewhere [25, 26]; the type of nutrient supplementation did not affect maternal anti-malarial antibodies, or susceptibility to malaria. Maternal haemoglobin at 36 gestation weeks (gw) and birth weight, gestational age and infant length at delivery were measured, and placental histology was examined.

Peripheral venous blood samples were collected at enrolment and at 36 gw. Only samples collected at enrolment with a paired 36 gw sample were selected for analysis. Samples were not available at 36 gw from women who did not attend this visit, women who had already delivered, and women from whom blood was not available for other logistical reasons. After exclusion of eight sets of twins, all available pairs of samples were assayed. Blood plasma was separated at each time point and stored frozen at −80 °C before shipping to Australia for measurements of anti-malarial immunity. Malaria parasitaemia was determined using blood film microscopy, rapid diagnostic test (RDT), polymerase chain reaction (PCR), and placental histology at delivery. Placental malaria (PM) was categorized as active infection (presence of pRBCs with or without haemozoin in monocytes and/or fibrin); past infection (haemozoin without pRBCs); or no infection, based on placental histology. Due to the low numbers of active placental infections (n = 31), only past placental infection and no infection were compared.

### Ethics approval

Ethical approval for the trial and the laboratory work was granted by the College of Medicine Research and Ethics Committee of Malawi, and by Tampere University Hospital Ethics Committee, Finland. The Melbourne Health Human Research Ethics Committee approved the laboratory work performed for the study.

### Parasite and cell culture

*Plasmodium falciparum* isolates CS2 and E8B were selected as representatives of placental-binding and non-placental-binding parasite isolates. CS2 binds to CSA expressed on the placenta while E8B binds to endothelial receptors CD36 and ICAM-1 [27, 28]. Parasite cultures were maintained as described in [29]. Pre-monocytic THP-1 cells were cultured for the opsonic phagocytosis assay and maintained as described in [30].

### Measurement of IgG and opsonizing antibodies to VSA expressed by placental-binding and non-placental-binding parasite isolates

Plasma samples from women who had samples available from study enrolment and at 36 gw were used to measure antibody levels. Flow cytometry based immunoassays were performed as previously described [25] to determine relative levels of naturally-acquired IgG to VSA expressed by the parasite isolates described above, and to measure levels of antibodies that opsonize pRBC for phagocytosis by THP-1 cells. Plasma was assayed at 1:20 dilution (IgG to VSA) or 1:10 (opsonizing antibodies). For both types of assay, antibody levels were reported as a percentage of the positive controls where the positive controls were a pool of human plasma with known high levels of antibody to VSA antigens. Positivity for each antigen was calculated as mean plus three standard deviations of the fluorescence intensity of 30 different negative controls.

### Measurement of IgG to merozoite antigens and schizont extract via fluorescent immunosorbent assay

Recombinant merozoite antigens MSP1 19kD, MSP2, MSP3, PfRh2 (construct PfRh2-2030) (6088–7584 bp) and EBA175 (region III-V) were prepared as detailed [25]. A fluorescent immunosorbent assay was designed to measure IgG to merozoite antigens as described in [25] and the IgG levels were reported as a percentage of the positive control, where the positive control was a pool of human plasma collected from individuals with immunity to malaria.

### Statistical methods

The outcome variables of interest included maternal haemoglobin levels (g/l, continuous) at 36 gw; past placental malaria (PM) compared to no placental malaria; birthweight (in grams, continuous variable); small for gestational age (SGA: binary variable defined as birthweight below the 10th centile on the United States national reference scale for fetal growth [31]), stunting: length-for-age Z-score (LAZ) <−2 at birth (binary variable); and small head circumference (HCZ <−2).

The selected covariates included gravidity, maternal age, HIV, bed net use, body mass index (BMI) at enrolment, malaria microscopy at enrolment, socioeconomic status (SES), study site, season at enrolment and supplementation group, and were recorded as described [25]. These covariates were chosen based on their effects on malaria antibody levels and pregnancy outcomes in the cohort, or their previously-demonstrated biological relevance to the association between antibody levels and pregnancy outcomes. Gravidity groups were categorized as primi, secundi and multigravidae. Maternal age was categorized as <21, 21–25, 26–30 and >30 years. Maternal BMI at enrolment was categorized into low (<18.5 kg/m^2^), normal (18.5–25 kg/m^2^) and increased (>25 kg/m^2^). The SES was calculated based on a household assets adjusted z-score (HHAZ) system [32], where a participant with a HHAZ below the median (z-score = −0.387) were considered as having a low SES. HIV, malaria infection, seasons at enrolment (rainy and dry) and ITN use were incorporated as dichotomous variables. Study sites (Lungwena, Malindi, Namwera and Mangochi) and supplementation groups (IFA, MMN and LNS) were used as categorical variables.

### Statistical analyses of the relationship between antibody levels and pregnancy outcomes after adjusting for covariates

Statistical analyses were performed using STATA 13.0 version (StataCorp LP, Texas, USA). Differences in the characteristics between the participants included and excluded in the study were determined by performing a Student’s *t* test for normally distributed variables or Mann–Whitney U test for non-normally distributed variables, or test of proportion for categorical variables.

Univariate and multivariate linear or logistic regression analysis were performed as appropriate to assess the relationship between antibody levels at enrolment and pregnancy outcomes adjusting for the covariates described above. Antibody levels were treated as continuous variables or as categorical variables following categorization into low, middle and high antibody tertiles.

## Results

### Study participants

Following exclusion of twins, antibody determination was performed for plasma samples collected from 1002 participants at enrolment who also had a sample at 36 gw. Among participants with samples at 36 gw, only 2.8% had preterm births.

Socio-demographic characteristics and birth outcome differences between iLiNS study participants who were included and excluded from the present analyses were determined (Table 1). Women included in the analyses were similar to those excluded in the analysis in age, HIV prevalence, SES and BMI. However women who were excluded had shorter pregnancies (because only women with 36 gestation week data available were included), more frequently delivered children with low birthweight, were more likely to be in their first pregnancy, and less likely to use ITNs compared to those who were included. Among included women, 10% had malaria infection detected by microscopy and 25% by PCR at enrolment (Table 1).

**Table 1:** Comparison of characteristics of the participants included and excluded in the analysis

Characteristic	Included (n = 1002)	Excluded (n = 377)	p value
Gestation weeks at delivery, mean (SD)	39.8 (1.5)	37.0 (4.9)	<0.0001*
Maternal age, years, mean (SD)	24.5 (5.8)	25.3 (6.1)	0.214
Gravidity, number (%)			
Primigravidae	199 (19.9%)	104 (27.6%)	0.002*
Secundigravidae	201 (20.1%)	80 (21.2%)	0.630
Multigravidae	600 (59.9%)	192 (50.9%)	0.003*
Low socio-economic status, number (%)	554 (55.7%)	162 (50.9%)	0.135
BMI at enrolment, median (IQR), kg/m^2^	21.6 (20.3, 23.5)	22.1 (20.4, 23.7)	0.063
HIV infected, number (%)	129 (13.0%)	52 (15.9%)	0.186
Maternal haemoglobin levels at 36 gw, median (IQR), g/l	110 (101, 120)	115 (106, 123)	0.052
Prevalence of anaemia at 36 gw (Hb <100 g/l), number (%)	200 (20.9%)	12 (14.5%)	0.165
Birthweight, mean (SD), g	3011 (412.8)	2791 (536.5)	<0.0001*
Prevalence of low birthweight, <2500 g	96 (10.3%)	51 (23.8%)	<0.0001*
Prevalence of SGA, number (%)	255 (30.6%)	54 (29.0%)	0.668
Microscopy at enrolment, number (%)	107 (10.7%)	39 (10.4%)	0.872
PCR positivity for malaria at enrolment, number (%)	245 (25.0%)	111 (30.2%)	0.054
PCR positivity for malaria at 36 gw, number (%)	92 (9.3%)	4 (5.1%)	0.212
Placental malaria			
Active infection	31 (3.8%)	11 (5.6%)	0.257
Past infection	293 (36.0%)	55 (28.1%)	0.037*
Uninfected	489 (60.1%)	130 (66.3%)	0.110
ITN use at enrolment, number (%)	399 (72.0%)	122 (59.5%)	0.001*

Data presented as mean (SD), median (IQR) or number (proportion of women as a percentage) for women included and excluded in the study, allowing for missing observations for each variable

*SD*, standard deviation; *IQR*, interquartile range; *BMI*, body mass index; *HIV*, human immunodeficiency virus; *Hb*, haemoglobin; *SGA*, small for gestational age; *PCR*, polymerase chain reaction

### Associations between malaria antibody at enrolment and maternal haemoglobin concentration at 36 weeks and birth weight

Median haemoglobin levels at 36 gw were 110 g/l (IQR 101–120; Table 1). After adjustment for covariates including gravidity and malaria at enrolment, there was a statistically significant positive association between antibodies measured before 20 gestation weeks to placental-binding VSA and haemoglobin concentration; with every 10% increase in antibody levels the haemoglobin concentration at 36 gw increases by 0.5 g/l (95% CI 0.05, 0.9; p = 0.029) for IgG to placental-binding VSA and 0.4 g/l (0.04, 0.8; p = 0.030) for opsonic phagocytosis activity of antibodies to placental-binding VSA (Table 2). The median levels of antibodies to placental-binding VSA and of opsonic phagocytosis activity of antibodies were 15.0 and 32.7% of positive controls, respectively. In contrast, antibodies to MSP1 19kD, MSP2, EBA175 and schizont extract were negatively associated with haemoglobin levels at 36 weeks (coefficients −0.5 to −1.3 g/l, 0.003 < p<0.032; Table 2). There was no statistically significant association between levels of antibodies to the non-placental-binding line, or to recombinant MSP3 or Rh2A9, and maternal haemoglobin concentration.

**Table 2:** Association between the participants’ malaria antibody levels at enrolment and their blood haemoglobin concentration at 36 gestation weeks and their child’s birthweight

Antibody type	Haemoglobin concentration (g/l)		Birthweight (g)	
	Coefficient (95% CI)	p value	Coefficient (95% CI)	p value
IgG to placental-binding VSA	0.5 (0.05, 0.9)	0.029*	−9.7 (−21.5, 2.1)	0.106
Opsonising antibodies to placental-binding VSA	0.4 (0.04, 0.8)	0.030*	2.0 (−9.0, 13.0)	0.721
Opsonising antibodies to non-placental-binding VSA	0.2 (−0.3, 0.6)	0.476	−3.1 (−16.4, 10.2)	0.651
MSP1 19kD	−0.7 (−1.3, −0.1)	0.018*	9.4 (−7.8, 26.6)	0.283
MSP2	−0.7 (−1.2, −0.1)	0.017*	5.2 (−10.3, 20.8)	0.507
MSP3	−0.2 (−0.7, 0.4)	0.592	−13.2 (−29.7, 3.3)	0.115
EBA175	−1.1 (−1.7, −0.4)	0.003*	5.3 (−14.0, 24.5)	0.589
PfRh2	−0.2 (−0.8, 0.5)	0.604	−8.6 (−27.3, 10.0)	0.364
Schizont extract	−0.5 (−0.10, −0.05)	0.032*	3.1 (−10.1, 16.2)	0.648

Analysis for each individual antibody was adjusted for gravidity, maternal age, HIV, ITN use, body mass index at enrolment, malaria microscope positivity at enrolment, socioeconomic status, study site, season at enrolment and supplementation groups. Results show increase in haemoglobin or birth weight per 10% increase in antibody

*95* *% CI*, 95% confidence interval; *IgG*, immunoglobulin G; *VSA*, variant surface antigens, *MSP*, merozoite surface protein; *EBA175*, erythrocyte binding homologue 175; *PfRh2*, *Plasmodium falciparum* reticulocyte binding homologue 2

In adjusted analyses, antibodies to none of the targets examined were significantly associated with birthweight; differences attributable to each 10% increase in antibody ranged up to 13 g (Table 2).

### Association between malaria antibody at enrolment and placental malaria, small for gestational age and low length-for-age

To examine relationships between antibody immunity and categorical outcomes, antibody responses were divided into three equal-sized groups, high, medium and low.

Because few women (31, 4%) had active placental malaria, the analysis compared only past PM (present in 36%) to no PM. In adjusted analyses, pregnant women in the high antibody tertile for MSP2 were more likely to have past PM than those in the low antibody tertile, OR 1.83 (95% CI 1.01, 3.31; p = 0.046) (Table 3). There were odds ratios of similar magnitude for high compared to low levels of opsonizing antibodies to the placental-binding and non-placental-binding parasite lines and high compared to low levels of antibody to schizont extract at enrolment but these associations were not statistically significant (Table 3).

**Table 3:** Association between antibody tertiles at enrolment and odds of past placental malaria

Antibody type	Past placental malaria			
	Middle compared to low antibody tertile		High compared to low antibody tertile	
	OR (95% CI)	p value	OR (95% CI)	p value
IgG to placental-binding VSA	1.20 (0.65, 2.22)	0.558	1.30 (0.70, 2.40)	0.402
Opsonising antibodies to placental-binding VSA	1.64 (0.91, 2.98)	0.102	1.82 (0.99, 3.36)	0.054
Opsonising antibodies to non-placental-binding VSA	1.31 (0.73, 2.35)	0.363	1.74 (0.97, 3.13)	0.065
MSP1 19kD	1.23 (0.69, 2.18)	0.488	1.27 (0.72, 2.25)	0.407
MSP2	1.47 (0.82, 2.64)	0.198	1.83 (1.01, 3.31)	0.046*
MSP3	0.59 (0.33, 1.05)	0.071	1.12 (0.65, 1.92)	0.691
EBA175	0.97 (0.55, 1.71)	0.913	1.14 (0.66, 1.99)	0.636
PfRh2	0.94 (0.54, 1.63)	0.818	1.05 (0.59, 1.85)	0.879
Schizont extract	1.51 (0.78, 2.92)	0.223	1.70 (0.90, 3.24)	0.104

Data presented as odds ratio (95% confidence interval). Multivariate logistic regression analysis performed to determine the risk of past PM between pregnant women in the middle antibody tertile compared to women in the low antibody tertile. Analysis adjusted for gravidity, maternal age, HIV, ITN use, body mass index at enrolment, malaria microscopy at enrolment, socioeconomic status, study site and supplementation groups

*OR*, odds ratio, *95* *% CI*, 95% confidence interval; *IgG*, immunoglobulin G; *VSA*, variant surface antigens, *MSP*, merozoite surface protein; *EBA175*, erythrocyte binding homologue 175; *PfRh2 Plasmodium falciparum* reticulocyte binding homologue 2

In adjusted analyses, antibodies to none of the targets examined were significantly associated with being born SGA (Table 4). Women in the middle tertile for opsonizing antibody to placental-binding VSA had a significantly higher risk of LAZ <−2, RRR 2.25 (95% CI 1.14, 4.42) than women in the low tertile group, but this was not observed in comparisons of high versus low antibody comparisons (OR 1.14 95%CI 0.53, 2.47) (Table 4). No other significant associations were found, and the OR for other associations was small.

**Table 4:** Association between antibody tertiles at enrolment and small for gestational age and low length-for-age (LAZ <−2)

Antibody type	Small for gestational age				Low length-for-age (LAZ <−2)			
	Middle compared to low antibody tertile		High compared to low antibody tertile		Middle compared to low antibody tertile		High compared to low antibody tertile	
	OR (95% CI)	p value	OR (95% CI)	p value	OR (95% CI)	p value	OR (95% CI)	p value
IgG to placental-binding VSA	0.74 (0.42, 1.31)	0.301	0.91 (0.52,1.60)	0.736	1.27 (0.62, 2.62)	0.513	1.05 (0.50, 2.21)	0.894
Opsonising antibodies to placental-binding VSA	0.92 (0.54, 1.57)	0.757	0.78 (0.45, 1.35)	0.369	2.25 (1.14, 4.42)	0.019*	1.14 (0.53, 2.47)	0.734
Opsonising antibodies to non-placental-binding VSA	1.34 (0.79, 2.28)	0.282	1.17 (0.67, 2.02)	0.584	1.29 (0.65, 2.56)	0.465	1.13 (0.56, 2.28)	0.735
MSP1 19kD	1.21 (0.71, 2.05)	0.476	0.93 (0.55, 1.59)	0.795	1.05 (0.54, 2.02)	0.889	0.75 (0.38, 1.48)	0.408
MSP2	1.12 (0.66, 1.89)	0.671	0.82 (0.48, 1.42)	0.486	0.97 (0.50, 1.90)	0.930	0.96 (0.48, 1.93)	0.918
MSP3	1.28 (0.75, 2.18)	0.358	1.65 (0.98, 2.80)	0.060	0.87 (0.44, 1.72)	0.682	1.28 (0.67, 2.46)	0.459
EBA175	1.62 (0.94, 2.75)	0.081	1.45 (0.85, 2.45)	0.172	0.88 (0.45, 1.72)	0.711	0.84 (0.43, 1.62)	0.608
PfRh2	1.26 (0.75, 2.13)	0.378	1.01 (0.59, 1.72)	0.979	1.18 (0.61, 2.29)	0.620	0.92 (0.46, 1.84)	0.815
Schizont extract	1.25 (0.68, 2.28)	0.469	1.44 (0.80, 2.61)	0.226	1.15 (0.54, 2.44)	0.724	1.27 (0.61, 2.63)	0.518

Data presented as odds ratio (95% confidence interval). Multivariate logistic regression analysis performed to determine the risk of giving birth to a child with SGA and LAZ <−2 among pregnant women in middle and high antibody tertile compared to women in the low antibody tertile. Analysis adjusted for gravidity, maternal age, HIV, bed net use, body mass index at enrolment, malaria microscopy at enrolment, socioeconomic status, study site, season at enrolment and supplementation groups

*LAZ*, length-for-age z-score; *OR*, odds ratio; *95* *% CI*, 95% confidence interval; *IgG*, immunoglobulin G; *VSA*, variant surface antigens, *MSP*, merozoite surface protein; *EBA175*, erythrocyte binding homologue 175; *PfRh2*, *Plasmodium falciparum* reticulocyte binding homologue 2

## Discussion

This study tested the hypothesis that measures of malaria antibody immunity at first antenatal visit could predict pregnancy outcomes in Malawian women enrolled in the iLiNS-DYAD-M clinical trial. In 1002 women, the study showed that IgG to VSA and antibodies promoting opsonic phagocytosis of pRBCs of placental-binding parasite isolates were associated with higher maternal haemoglobin levels at 36 gw. These measures of pregnancy-specific malaria immunity were not significantly associated with birthweight or SGA in adjusted analyses. Antibodies to other malaria antigens assessed either demonstrated no significant associations or negative associations with the pregnancy outcomes assessed. Antibodies to merozoite antigens MSP1 19kD, MSP2, EBA175 and schizont extract were associated with lower haemoglobin concentration at 36 weeks and antibodies to MSP2 were associated with an increased likelihood of past PM. The study was performed in women receiving recommended doses of IPTp and ITNs, and investigated potential additional benefits of antibody immunity on outcomes.

The positive associations between both IgG and opsonizing antibodies to placental-binding isolate VSA and haemoglobin levels at 36 gw are in agreement with previous observations in pregnant women from Blantyre, Malawi [33, 34] and Kilifi, Kenya [35], although two of these studies examined antibody responses only at term. By contrast, IgG to VSA of a placental-binding isolate, measured (like this study) in samples collected in first half of pregnancy, was not associated with haemoglobin concentrations in a previous clinical trial in Lungwena, Malawi [18]. In that study, antibody responses were measured in 549 women (about half the present study), and the study lacked statistical power to detect modest associations. Maternal anaemia may increase the risk of preterm births [36] and interferes with fetal growth causing LBW [2, 37]. Although the haemoglobin concentration was only 0.4–0.5 g/l higher with every 10% increase in antibody levels (so a woman with antibody level 100% of controls is expected to have a haemoglobin 5 g/l higher than a matched woman with no antibody) the positive association observed in the current cohort suggests a potential role of these antibodies in preventing malaria-induced anaemia.

The associations between levels of antibodies to some merozoite proteins or to schizont extract and risk of anaemia suggest that some of these antibodies may be acting as markers of previous malaria infection, rather than predictors of better pregnancy outcomes. Antibodies to merozoite antigens are well known to increase with exposure [38]. Repeated malaria episodes will lead to cumulative destruction of RBCs and lower haemoglobin levels. SES was strongly associated with malaria risk (likely to be correlated with exposure, e.g. through poor housing) and was adjusted for in the analysis, but there may be other factors that affected malaria and anaemia risk in this group which were not identified and so not adjusted for, such as location and characteristics of residence, which may affect malaria exposure, but might also be markers of poverty and/or nutritional insecurity.

There was a weak association between antibodies to MSP2 and past placental malaria, and similar trends for antibody to schizont antigen and opsonizing antibody to both placental-binding and non-placental-binding parasite lines. Past placental malaria is characterized by persistent haemozoin deposits in fibrin in the placenta with no pRBCs [39], a sign of infection that has been cleared prior to delivery. Women with high antibody levels at study entry likely live in environments where they are at great risk of malaria infection, and this higher risk may persist in pregnancy despite use of IPTp and ITNs, and so will increase the likelihood they have experienced placental malaria at some point in pregnancy. Placental blood circulation is established at around 9–12 weeks gestation [40]. Infections occurring before study enrolment may increase antibodies to MSP2, while infections present at any time after 9–12 weeks’ gestation may affect the placenta, but will be cleared by IPTp leaving residual pigment. It is likely that the same women are at higher risk of malaria over both periods.

Antibodies to placental-binding VSA were not significantly associated with improved newborn anthropometrics unlike previous reports [9, 35, 41]. In previous reports the mothers were secundigravidae [9], secundigravidae with placental malaria and HIV [41] or women with chronic placental infection [35] and antibody levels were measured at delivery rather than in early pregnancy. Rather than demonstrating population-wide associations between malaria antibodies and birthweight (as studied here), most published studies have been restricted to women of a specific gravidity or HIV and/or malaria infection status [12, 30, 33]. Further prospective studies of these high-risk groups for associations between immunity and outcome may be indicated.

There was an unexpected negative association between opsonizing antibody to the placental-binding parasite line and newborn length, seen when women in the middle and low antibody groups were compared. The cause of this is not clear, and there was no similar trend when women with high and low antibody response were compared, nor was there a similar association between levels of IgG antibody to VSA of the placental-binding line and newborn length.

Endpoints such as maternal haemoglobin, birth weight, SGA and newborn length are multifactorial in aetiology, and estimating the proportion of each attributable to malaria is challenging. Study participants received protection against malaria with two doses of IPTp with sulphadoxine pyrimethamine and ITNs, limiting their exposure to malaria after enrolment, decreasing their risk of anaemia and LBW, and improving birth weights [42]. Only 3% of women had active malaria infection on placental histology, and malaria prevalence by RDT was 8%, a decrease from 23% at first antenatal visit (M Nkhoma, in submission). Malaria in later pregnancy was associated with low birth weight and decreased fetal size (M Nkhoma, in submission), but in settings where malaria prevalence is low (such as when malaria transmission falls), the attributable fractions of low birth weight and other outcomes due to malaria may be low, and it may therefore become increasingly difficult to identify immune responses to malaria that protect against adverse pregnancy outcomes.

Strengths of the study included a large sample size, of over 1000 women; analysis of a range of antibody measures; and extensive clinical data. Linear and logistic regression analyses were used to adjust for factors associated with the input and/or dependent variables of interest. Study limitations included few women with preterm delivery (because only women with a 36 gw visit were included), preventing an investigation of whether antibody protects against preterm delivery; measurement of antibody responses to single parasite isolates of each phenotype, and single variants of each of a modest number of merozoite proteins; and measurement of a single functional role of antibodies (opsonization of pRBC for phagocytosis). Apart from preterm delivery and associated lower birth weights, other characteristics more common in excluded women included being primigravid, and higher rates of malaria infection by PCR at enrolment. This may have limited our power to find protective associations. Quantitating antibody to other placental-binding parasite isolates, [43] and understanding other antibody functions, such as merozoite opsonization [44] or complement fixation [45] could be beneficial in future studies. HIV was included as a co-factor, but helminth and bacterial infections which are prevalent among Malawian pregnant women [46–48] were not evaluated, which may have influenced certain analyses such as association with haemoglobin concentration. To dissect the determinants of aetiologically-complex pregnancy outcomes such as maternal haemoglobin, birth size, or preterm delivery, a broad range of comorbid conditions may need to be addressed.

The study findings suggest that antibody to pregnancy-specific parasite isolates, measured using two different approaches, may be associated with protection from malarial anaemia, whereas antibodies to merozoite-specific antigens are not. Further studies that prospectively evaluate pregnancy-specific and more general measures of malaria antibody, under a range of transmission settings, will be required to determine whether one or more measures may identify women at risk with sufficient sensitivity and specificity to be considered as a screening tool for high-risk pregnancies in the future. The lack of evidence that naturally-acquired antibodies to one placental binding parasite line were associated with improved fetal growth may reflect the relatively modest impact of malaria on birth weight in this cohort. Demonstration that induction of antibodies by a VAR2CSA based vaccine improves pregnancy outcomes would require careful selection of target populations in which malaria is a major contributor to adverse pregnancy outcomes.

## Abbreviations

BMI: body mass index
CSA: chondroitin sulphate A
EBA: erythrocyte binding antigen
gw: gestation weeks
HCZ: head circumference z score
HHAZ: household assets z score
HIV: human immunodeficiency
IFA: iron and folic acid
IPTp: intermittent preventive treatment in pregnancy
IQR: interquartile range
ITN: insecticide treated net
LAZ: length-for-age z score
LBW: low birth weight
LNS: lipid-rich nutrient supplement
MMN: multiple micronutrients
MSP: merozoite surface protein
OR: odds ratio
PCR: polymerase chain reaction
PfRh2: *Plasmodium falciparum* reticulocyte binding homologue 2
PM: placental malaria
pRBC: parasitized red blood cell
SES: socioeconomic status
SGA: small for gestational age
VSA: variant surface antigen
